# Internet and Face-to-face Cognitive Behavioral Therapy for Postnatal Depression Compared With Treatment as Usual: Randomized Controlled Trial of MumMoodBooster

**DOI:** 10.2196/17185

**Published:** 2021-12-08

**Authors:** Jeannette Milgrom, Brian G Danaher, John R Seeley, Christopher J Holt, Charlene Holt, Jennifer Ericksen, Milagra S Tyler, Jeff M Gau, Alan W Gemmill

**Affiliations:** 1 Parent-Infant Research Institute Heidelberg Repatriation Hospital Heidelberg Heights Australia; 2 Melbourne School of Psychological Sciences University of Melbourne Parkville Australia; 3 University of Oregon Eugene, OR United States; 4 Influents Innovations Eugene, OR United States; 5 Australian College of Applied Psychology Melbourne Australia

**Keywords:** postnatal depression, postpartum depression, postnatal anxiety, postpartum anxiety, cognitive behavioral therapy, internet intervention, web-based intervention, randomized controlled trial, online intervention, treatment, mobile phone

## Abstract

**Background:**

Previous research has confirmed that symptoms of postnatal depression (PND) can be ameliorated through internet-delivered psychological interventions. Advantages of internet-delivered treatment include anonymity, convenience, and catering to women who are unable to access face-to-face (FTF) treatments. To date, no research has examined the efficacy of such interventions compared directly with FTF treatments in women clinically diagnosed with PND.

**Objective:**

This study aims to compare the efficacy of one of the first web-based cognitive behavioral therapy (CBT) interventions (internet CBT+coach calls) for PND (*MumMoodBooster* [MMB]) with FTF-CBT in a randomized controlled trial (RCT).

**Methods:**

In this study, 116 postnatal women with a *Diagnostic and Statistical Manual for Mental Disorders, Fourth Edition* (*DSM-IV*) diagnosis of major or minor depression were randomized to MMB (39/116, 33.6%), FTF-CBT (39/116, 33.6%), or a treatment-as-usual (TAU) control condition (38/116, 32.8%). Diagnostic status was determined at baseline and at 21-week follow-up using the Structured Clinical Interview for the DSM-IV. Severity of anxiety and depressive symptoms was evaluated using the Depression Anxiety Stress Scales and the revised Beck Depression Inventory at baseline, 12-week follow-up (after treatment), and 21-week follow-up.

**Results:**

Of the 116 participants, 107 (92.2%) had a diagnosis of major depression at baseline. Rates of remission from a major or minor depressive episode at 21 weeks in both the FTF-CBT and MMB groups were superior to that of the TAU group (56.6% and 47.7% less likely to be depressed, respectively) and they were not significantly different from each other. Although remission rates differed between TAU and FTF-CBT, growth models showed that, in terms of symptom reduction across time, the FTF-CBT treatment was not significantly better than TAU. By comparison, MMB was statistically superior to both TAU and FTF-CBT in reducing symptoms of depression, anxiety, and stress from baseline to the 21-week follow-up (large and moderate effect sizes). Thus, after 21 weeks, the average symptom scores for depression and anxiety of women receiving MMB were approximately half those of women in both the TAU and FTF-CBT groups.

**Conclusions:**

In this RCT, MMB was at least as effective as FTF-CBT in achieving remission from a diagnosed PND episode. MMB was superior to TAU and FTF-CBT in encouraging and maintaining reduction of symptom severity over the 21-week follow-up for depressed postnatal women. These findings replicate results of prior studies on MMB that showed clinically significant improvements in depressive symptoms, and they provide direct empirical support that internet-delivered treatment for depressed postnatal women is a viable alternative to FTF treatment. The generalizability of the results needs to be examined in future research, as RCTs of internet-based versus FTF treatments necessarily involve a subset of people who are willing to undertake either modality of treatment.

**Trial Registration:**

Australia and New Zealand Clinical Trials Registry (ANZCTR) ACTRN12613000881730; https://anzctr.org.au/Trial/Registration/TrialReview.aspx?id=364683&isReview=true

## Introduction

### Background

A substantial proportion of new mothers suffer from postnatal depression (PND). A meta-analysis by Gavin et al [1] estimated a point prevalence of 12.9% at 3 months postpartum. Left untreated, PND has significant deleterious effects on the mother (eg, absence of well-being, feelings of lack of competence in the mothering role, and interactional difficulties with her infant), her partner (eg, partner’s mental health and marital functioning or couple relationship problems), and her child (eg, diminished cognitive and psychosocial development) [2-5]. Children of mothers with PND are at an elevated risk of mental health difficulties during adolescence [6].

A considerable body of research supports the effectiveness of cognitive behavioral therapy (CBT) interventions for depression in clinical settings [7,8]. Approximately two-thirds of individuals in CBT trials are no longer diagnosed with depressive disorders at follow-up [8], and CBT also reduces the risk of relapse [9], particularly in cases of mild-to-moderate depression [10]. A range of treatment approaches is effective specifically for PND, and these have been systematically reviewed by Dennis and Hodnet [11] and Cuijpers et al [12]. Treatment approaches supported by positive research findings include CBT [13], counseling [14], pharmacotherapy [15], and interpersonal psychotherapy [16]. Milgrom et al [13] successfully adapted CBT treatments to the needs of new mothers with PND. These treatments were developed to be sensitive to specific needs during the perinatal period, for example, less time commitment to homework, techniques of relaxation on the run, and inclusion of women’s partners where appropriate [13].

Reviews of the available evidence [17-19] have concluded that self-guided internet interventions are beneficial to depressed individuals but that the largest effect sizes are achieved when these are combined with guided human support, typically delivered via telephone or email contact. Such support can increase adherence to internet-delivered mental health treatments [20,21] and may also provide a *safety net* for individuals who may need additional help should their situation worsen. Alliance with a *trustworthy* coach is central to this model, and a number of studies have shown that a strong web-based working alliance can be achieved in guided internet-delivered treatments for posttraumatic stress disorder and depression [22,23]. The emerging picture is that very encouraging therapeutic effects can be achieved through structured internet programs, supported by low-intensity human guidance (typically <3 contact hours in a 6-week program) [17-19].

### Effectiveness of Internet Psychological Interventions

The balance of existing evidence [18,24-26] suggests that psychological treatments delivered via the internet can be as effective as traditional face-to-face (FTF) approaches [27,28]. Moderate-to-large effects are generally reported [27], although it should be noted that, in many studies, the typical counterfactuals are waitlist controls and treatment as usual (TAU). For depression and anxiety in particular, the efficacy of internet-based treatment has been demonstrated relative to control conditions in populations with elevated symptoms and increasingly in clinically diagnosed groups [25,29-34]. Multiple meta-analyses and systematic reviews of internet interventions have confirmed clinically and statistically significant improvements in symptoms compared with TAU [25,27,34-36].

### The Need for Internet Interventions Specific to PND

PND is undertreated: fewer than half of perinatal women seek help even when identified as depressed [37]. Barriers to clinic-based treatment uptake include fear of stigma, being perceived as a *bad* mother, feelings of failure, poor understanding of depression or the available help, concern about medication being passed through breastmilk [38], and difficulty attending because of the demands of the new baby [39]. Internet psychological interventions may offer advantages in this population. The perception that the internet is somewhat anonymous may assist in circumventing stigma [40]. Similarly, better accessibility may be achieved with a treatment program available from home. The flexibility of being able to review program content at any time on any day in a convenient location may help in minimizing childcare logistics and fitting in with changing and sleep-disrupted family schedules. Such interventions could then potentially provide psychological treatment to many women with PND who otherwise would not access support.

Spurred by these possibilities, a number of such internet interventions have been developed to address maternal perinatal depression. Beginning with our own work [41], the delivery of cognitive and behavioral internet interventions for perinatal symptoms of depression has emerged as a major focus. Several feasibility studies and randomized trials using CBT and behavioral activation have shown significant effects on postnatal depressive and anxiety symptoms, including those in the clinically severe range [42-46]. Compared with a TAU comparison group, Milgrom et al [43] reported a 4-fold increase in remission from a diagnosed depressive disorder using the Structured Clinical Interview for the DSM-IV (SCID-IV) at 3 months after enrollment in the 6-session internet CBT intervention named MumMoodBooster (MMB; the US program version is named MomMoodBooster). O’Mahen et al [44,45,47] reported similar reductions in depressive symptoms with a 12-session intensive coaching behavioral activation therapy (the Net Mums program). Laughnan et al [48,49] have shown encouraging short-term effects for their brief, transdiagnostic internet intervention (MUMentum postnatal) that targets symptoms of both depression and anxiety. These internet interventions vary considerably in length and in the amount and nature of guidance or support offered (ranging from extended contacts with professional therapists to brief, low-intensity *technician* or coach support). The emerging picture suggests that internet interventions may help overcome several important barriers to seeking treatment in perinatal populations, and they have the potential to offer a scalable alternative for the delivery of psychological treatments.

Meta-analyses suggest that internet interventions have achieved medium-sized effects in reducing perinatal depressive symptoms, usually in comparison with TAU or waitlist control conditions [50-52]. To date, no published study has examined the efficacy of an internet intervention versus FTF psychological treatment designed specifically for postnatal women with diagnosed depressive disorders.

### Aims of This Research

This study aims to evaluate remission from clinical depression and reduction of depressive and anxiety symptom severity among a sample of postnatal women with diagnosed depression. We aim to evaluate the relative efficacy for women randomly assigned to (1) MMB, a guided version of an internet intervention for PND; (2) a validated FTF-CBT program for PND; and (3) TAU. The secondary aim of this randomized controlled trial (RCT) is to compare the 2 active conditions with respect to symptom trajectory, stress, and process measures of treatment engagement, competence in the mothering role, marital functioning, acceptability, and satisfaction.

Our a priori hypothesis was that women allocated to either of the 2 active conditions (MMB and FTF-CBT) would have significantly higher remission rates, greater reductions in depression and anxiety symptoms, and greater improvement in secondary outcomes compared with the TAU condition but that MMB and FTF-CBT would not differ significantly from each other.

## Methods

### Experimental Design

This study was a parallel 3-group RCT involving 116 participants and consistent with the CONSORT standards [53,54]. The trial was prospectively registered in the Australia and New Zealand Clinical Trials Registry (trial registration number ACTRN12613000881730) and was approved by the human research ethics committee of Austin Health, Melbourne (approval number H2013/04972).

### Procedure

Screening and recruitment were conducted via maternal and child health centers in rural and metropolitan Victoria, Australia, and by localized advertising on the internet, in newspapers, and on the radio. The project was conducted between August 2014 and November 2017.

Inclusion criteria were determined in 2 phases. Phase 1 criteria included the following: Edinburgh Postnatal Depression Scale [55] scores of 11-25, aged ≥18 years, 6 weeks to 1 year postpartum; home internet access, familiarity with internet and email, and able and willing to give informed consent that included agreeing to be assigned to any of the 3 experimental conditions. Women who scored >0 on item number 10 of the Edinburgh Postnatal Depression Scale (thoughts of self-harm) were asked follow-up questions to ascertain intentionality, plan, lethality, access to means, and history of suicide attempts. Women deemed to be at risk of suicide were excluded and referred to receive immediate crisis attention.

Women found eligible in the screening phase 1 were subsequently assessed in phase 2 by a clinical psychologist or a provisional psychologist in a phone-administered SCID-IV [56]. Prospective study participants were excluded if they met any of the following exclusion criteria in screening phase 2: current substance abuse, manic or hypomanic symptoms or depression with psychotic features meeting the *Diagnostic and Statistical Manual of Mental Disorders, Fourth Edition* (*DSM-IV*) criteria, posttraumatic stress disorder, risk of suicide, and under current treatment for depression (medication or psychotherapy). Women who received a diagnosis of major or minor depressive episodes and who met all other eligibility criteria were invited to participate. Women who gave written consent and completed the baseline assessment were then randomized to a study condition.

### Randomization

Women were randomized in a 1:1:1 ratio to 1 of the 3 conditions: internet-based CBT with telephone support (MMB; 39/116, 33.6%), individual FTF-CBT (39/116, 33.6%), or TAU (38/116, 32.8%). An automated permuted block (block sizes of 3, 6, and 9) allocation schedule was pregenerated by a computer by an independent technician. Allocation concealment was ensured by a central, computer-automated administration. Upon completion of baseline questionnaires, the system assigned the participants to 1 of the 3 conditions. Their allocation was subsequently revealed to them in a phone call. Given the nature of the treatment conditions, allocation could not be concealed from participants beyond the point of allocation.

### Experimental Conditions

#### MumMoodBooster

MMB is designed to deliver content that is similar to FTF depression treatment, with tailored, interactive activities used to address individual issues and engage women. Support from a telephone coach is intended to encourage women to use and complete the program. The initial steps of the program provide explicit direction, whereas the latter steps encourage participants to assume increasingly greater responsibility for managing their own plan for change.

MMB uses tunnel information architecture [41,57] with step-by-step guidance through 6 sessions, with a new session becoming available for use every week. Session content is similar to that found in traditional FTF-CBT treatment using a scaffold approach that builds upon concepts presented in the previous sessions. MMB’s charting function was designed to help participants see the functional relationship between mood and activity levels—a key therapeutic concept in CBT that can be maximized in an internet program and, therefore, an important functionality in this program. Information from past sessions is used to reinforce gains made, and program content is tailored to individual issues with the provision of ipsative feedback [58]. A printable summary describes the key content covered in each session and a tailored list of recommended home practice activities. MMB includes content designed to enhance participant self-efficacy to accomplish recommended strategies as well as text, audios, and videos to engage participants in a program with some personalized elements. In recognition of the important role of partners and paternal depression in the treatment process of PND [59], an article in the MMB library entitled *You and Your Partner* provides content relevant to partners of women with PND. In addition, MMB enables participants to choose whether to invite their partner to use a free-standing partner support website with a separate user log-in. The MMB library also includes articles on relaxation, problem solving, and getting support for parenting (for further details on the development of the program, see the study by Danaher et al [41]). As described in earlier publications [42,43,60,61], to encourage optimal engagement and resulting behavior change [57], although the intervention can be accessed and used on smartphones, MMB was designed and optimized for use on desktop computers, laptops, and tablets and designed to function on popular browsers for both Windows PCs and Mac computers.

Weekly low-intensity telephone coaching support (30 minutes maximum per week) was provided. Rather than providing therapy per se, coaches were instructed to reinforce participant progress, encourage program use (practice of strategies and completion of tasks), and introduce the themes of upcoming sessions. In an initial welcome call, the assigned MMB coach explained the MMB core structure, additional library articles, and partner website. Coaches were able to access a secure administrative website to review each participant’s program use to help tailor their support. Coaching call fidelity was facilitated by the use of a manualized script and a session-by-session checklist. Coaching calls were provided over a period of 9 weeks, which enabled participants to work through the 6 sessions at <1 session per week and allowed for the rescheduling of up to 3 missed coach calls.

#### The FTF-CBT Condition

Women in the FTF-CBT condition were scheduled to receive weekly individualized CBT therapy from an experienced psychologist who followed a detailed, scripted manual for delivery of the *Getting Ahead of Postnatal Depression* program [13,62]. This is a manualized 9-session CBT-based program whose core content has been shown to be effective in several RCTs [13,63,64], and this was drawn upon in the original design of MMB. The program addressed maternal mood (depression and anxiety), behavioral activation, cognitive strategies, self-esteem, relaxation (*relax on the run*), getting support, and dealing with partner issues. The program included 1 additional session that involved both participants and their partners [62].

After screening was completed on the web, the research protocol was explained to potential recruits and following a SCID-IV interview, if randomized to FTF-CBT, participants visited their general practitioner (GP) with a referral letter from the study to arrange treatment with a suitable local psychology provider located by the research team. Psychologists kept session-by-session checklists of items covered in the FTF-CBT program. A report was also sent to the GPs of participants in the other 2 conditions; however, their GPs were not actively encouraged to connect their patients with a local psychology provider.

#### The TAU Condition

Women in the TAU condition were referred back to their GP supplemented with a written summary of their diagnostic assessment. Support and referral to other services could then occur as necessary as typically occurs in Australia when specialized programs are not available.

### Measures

Measures were collected primarily using web-based questionnaires, although phone calls were also used, as described in the following section (Table 1). All participants received automated email prompts reminding them to complete the web-based assessments. Participants were given a modest reimbursement in consideration of their time spent in completing questionnaires and assessments at the 12-week posttest and 21-week follow-up: Aus $20 (US $14.90) and Aus $35 (US $26), respectively.

**Table 1:** Measures and collection time points.

Measure		Baseline enrollment	Safety monitoring			Posttest, week 12	Week 16 (safety monitoring)	Follow-up, week 21
			Week 3	Week 5	Week 9			
**Depressive symptoms**								
	Structured Clinical Interview for DSM-IV^a^	✓^b^						✓
	Beck Depression Inventory	✓				✓		✓
	Patient Health Questionnaire	✓	✓	✓	✓	✓	✓	✓
**Anxiety symptoms**								
	Depression Anxiety Stress Scale	✓			✓	✓		✓
**Perceived stress**								
	Depression Anxiety Stress Scale	✓			✓	✓		✓
**Marital functioning**								
	Dyadic Adjustment Scale	✓				✓		✓
**CBT^c^ skills**								
	Negative thinking: Automatic Thoughts Questionnaire	✓				✓		✓
	Behavioral activation: Behavioral Activation for Depression Scale	✓				✓		✓
**Maternal self-efficacy**								
	Parenting Sense of Competence Scale Maternal Self-efficacy subscale	✓				✓		✓
**Monitoring and safety measures**								
	Risk management protocol	✓	✓	✓	✓	✓	✓	✓
	Treatment satisfaction							✓
	Use of other supports or treatments							✓

^a^DSM-IV: Diagnostic and Statistical Manual of Mental Disorders, Fourth Edition.

^b^Measurement collected.

^c^CBT: cognitive behavioral therapy.

#### Primary Outcome Measures

Trained diagnostic interviewers blinded to treatment allocation conducted the SCID-IV [56] by phone to determine a *DSM-IV* diagnosis of major depression or minor depression [65]. To measure the severity of depression, we used a web-based version of the revised Beck Depression Inventory (BDI-II) [66,67], a well-validated, 21-item clinical instrument that measures cognitive, affective, and physiological factors. The BDI-II has been used in numerous studies involving perinatal women [68-70] and has been validated against diagnostic criteria in perinatal populations [69]. The 9-item Patient Health Questionnaire (PHQ-9) [71] was used as a serial measure of depression for trajectory analysis. The PHQ-9 measures depressive symptoms in the past 2 weeks; it (1) is well-validated [71,72] (including with perinatal women [73]), (2) has high test–retest reliability [74], (3) has high internal consistency (α=.86) [75], and (4) is shown to be sensitive to changes in response to treatment [76]. Participants’ anxiety symptom severity was measured using the 7-item Anxiety Scale of the Depression Anxiety Stress Scale, 21-item (DASS-21) [77,78]. The DASS-21 uses a 4-point scale to describe agreement with statements over the past week and is rated as 0=did not apply to me at all, never; 1=applied to me to some degree or some of the time, sometimes; 2=applied to me to a considerable degree or a good part of the time, often; and 3=applied to me very much or most of the time, almost always. The sum of responses to the Anxiety Scale is scored as normal (0-7), mild (8-9), moderate (10-14), severe (15-19), and extremely severe (>20). The DASS-21 has good concurrent validity with other established anxiety scales [77].

#### Secondary Outcome Measures

##### CBT Skills: Negative Thinking

Participants completed the 30-item Automatic Thoughts Questionnaire (ATQ) [79]. The ATQ asks respondents to rate their agreement with a series of statements (eg, “My life is a mess”) using a 5-point scale (1=not at all to 5=all of the time). The maximum ATQ score is 150.

##### CBT Skills: Behavioral Activation

Participants completed the 25-item Behavioral Activation for Depression Scale [80], which measures changes in activation, avoidance or rumination, work or school impairment, and social impairment. Respondents rated their agreement with a series of statements (eg, “I stayed in bed for too long even though I had things to do”) on a scale of 0-6. The maximum Behavioral Activation for Depression Scale score is 150.

##### Maternal Self-efficacy

Drawing upon Bandura’s theoretical work, Wittkowski et al [81] described parenting self-efficacy as a parent’s belief in their ability to perform the parenting role successfully. To measure this, we used the Maternal Self-efficacy subscale of the Parenting Sense of Competence Scale [82], which asks respondents to rate the extent of their agreement with 7 items relating to self-perception of knowledge and competence in the mothering role. Each statement (eg, “I honestly believe I have all the skills necessary to be a good mother to my baby”) is rated from 1 (strongly disagree) to 6 (strongly agree). The maximum score is 42.

##### Marital Functioning

Women’s relationships with their partners were assessed using the 7-item Dyadic Adjustment Scale [83]. The general satisfaction score was calculated as the sum of all items. The maximum score is 36.

##### Treatment Satisfaction and Helpfulness

Participants in the MMB and FTF-CBT conditions were asked to rate their satisfaction with their assigned treatment as well as a set of select treatment components using a 4-point scale (0=not at all satisfied, 1=somewhat satisfied, 2=moderately satisfied, and 3=very satisfied). MMB participants were also asked to rate the helpfulness of phone coach calls using a 4-point scale (0=not at all helpful, 1=somewhat helpful, 2=moderately helpful, and 3=very helpful).

##### Use of Other Supports or Treatments

Participants were asked to complete a checklist of other support services and treatments they used during the study interval.

##### Participant Engagement

MMB use (overall number and duration of discrete visits from enrollment to the end of the follow-up assessment) was tracked continuously and unobtrusively via database flags and analysis of server activity logs (see our earlier MMB trial publications for more details [41-43]).

Coaches in the MMB condition and psychologists in the FTF-CBT condition recorded data on participant session completion on session-by-session compliance checklists itemizing the elements of each session plan covered. A subset of coaching calls and FTF-CBT therapy sessions were audio recorded (with permission) to facilitate fidelity checks.

All measures were collected at baseline, 12-week posttest, and 21-week follow-up, except for the SCID-IV diagnostic assessment, which was not conducted at 12 weeks. In addition, the PHQ-9 was also monitored during safety calls at weeks 3, 5, 9, and 16 after enrollment. The follow-up time point of 21 weeks was selected to reflect 3 months of posttreatment completion (with MMB and FTF-CBT treatment taking 9 weeks to complete).

### Safety Monitoring

Symptom severity and safety of participants in each of the RCT conditions were monitored in calls for all conditions using the PHQ-9. A written risk management protocol, based on the successful approach of Simon et al [84], was initiated if any risk of harm to self or infant or marked deterioration of depressive symptoms was indicated. These assessments of symptom severity and safety were scripted and accomplished on the phone by trained research staff who were blind to the respondents’ treatment allocation. In the 2 intervention conditions, weekly contact with therapists and coaches enabled additional monitoring of depression severity and safety.

### Data Analysis

Depression remission was assessed using the evidence-based definition of Frank et al [85], which requires a person to be asymptomatic (defined as no longer meeting the criteria for the disorder and having ≤2 symptoms for ≥2 weeks).

Categorical outcomes (eg, diagnostic status based on the SCID-IV) were analyzed using contingency tables and logistic regression; survival analysis (Cox regression and Kaplan–Meier product limit) was used to predict time-to-remission of the index depressive episode using the results from follow-up interviews. Continuous outcomes (eg, symptom severity) were analyzed using random-effects regression models, accommodating time-independent and time-dependent covariates, fixed and random factors, and incomplete data.

Random-effects growth models were estimated from baseline (the intercept) to the 21-week follow-up using the PROC MIXED procedure in SAS 9.2 (SAS Institute). The growth models were estimated with full information maximum likelihood and an unstructured variance/covariance matrix, and the intercepts and slopes were allowed to vary. First, unconditional means models were run for each outcome and the estimated and observed data plotted. Next, linear conditional growth models were run with time coded in weeks since the pretest assessment. All primary analyses to address major hypotheses adhered to intention-to-treat principles, and 3 a priori comparisons were examined: MMB versus TAU, FTF-CBT versus TAU, and MMB versus FTF-CBT (for each comparison, the first group was the reference group). The condition×time interaction is a test of the efficacy of the program for each a priori comparison. The effect size for the condition×time interaction was computed as a d-statistic equivalent [86].

Descriptive statistics and plots were used to screen all outcomes for normality and outliers. No serious violation of normality was found, with the exception of anxiety at 12 and 21 weeks, which showed a preponderance of zeros. During modeling, anxiety scores were examined in their normal metric and also log transformed to better approximate normality. A participant was flagged as an outlier on anxiety and stress at the 12-week assessment. During modeling, the outlier was evaluated for its impact on change over time.

### Clinical Significance, Power, and Sample Size

An a priori consideration of power suggested that for the survival analysis predicting time-to-remission from the index episode, with a 2-tailed α of .05, n=70 per condition would yield 80% power to detect a small effect size (hazard ratio 2.0). For the measure of depressive symptoms (BDI-II), data from previous work by Milgrom et al [13] provided a baseline value=23, σ=8.09. A difference of 6.5 takes scores below the threshold of *clinical* depression (ie, a score <17 points). With 80% power at α=.05, the required n=15.7×(8.09/6.5)^2^=24.3, which rounds up to 25. Taking into account a prudent noncompliance rate of 30%, the adjusted sample size, n* = 25 / (1 − 0.3)^2^ = 51, which rounds up to 55. Thus, an estimated n=70 per condition yielded sufficient power to detect a small between-group effect size and a minimum clinically important difference in the primary measure of symptom severity. This sample size was not achieved within the funding period; however, post hoc analysis of power with the sample size achieved confirmed sufficient power to address the minimum clinically important difference for the primary outcomes. We had sufficient power (>80%) to detect significant group differences (*P*<.05) in our primary outcomes. We used a simulation approach with 1000 replications using the likelihood ratio test [87] and implemented it in SAS. We estimated the power to detect condition effects for changes in the primary outcomes. Unlike the a priori power estimates, this post hoc power estimation included observed parameter estimates (eg, sample size, effect size, and correlations between intercept and slope). Thus, this study was sufficiently powered to detect group differences between MMB and TAU for medium to large effects.

### Depressive Symptom Trajectory

At baseline and during the routinely scheduled safety monitoring calls (at weeks 3, 5, 9, 12, and 21), all participants completed the 9-item PHQ-9 over the telephone. These data were used to calculate the trajectory of depressive symptom changes over time.

## Results

### Sample

**Figure 1:**
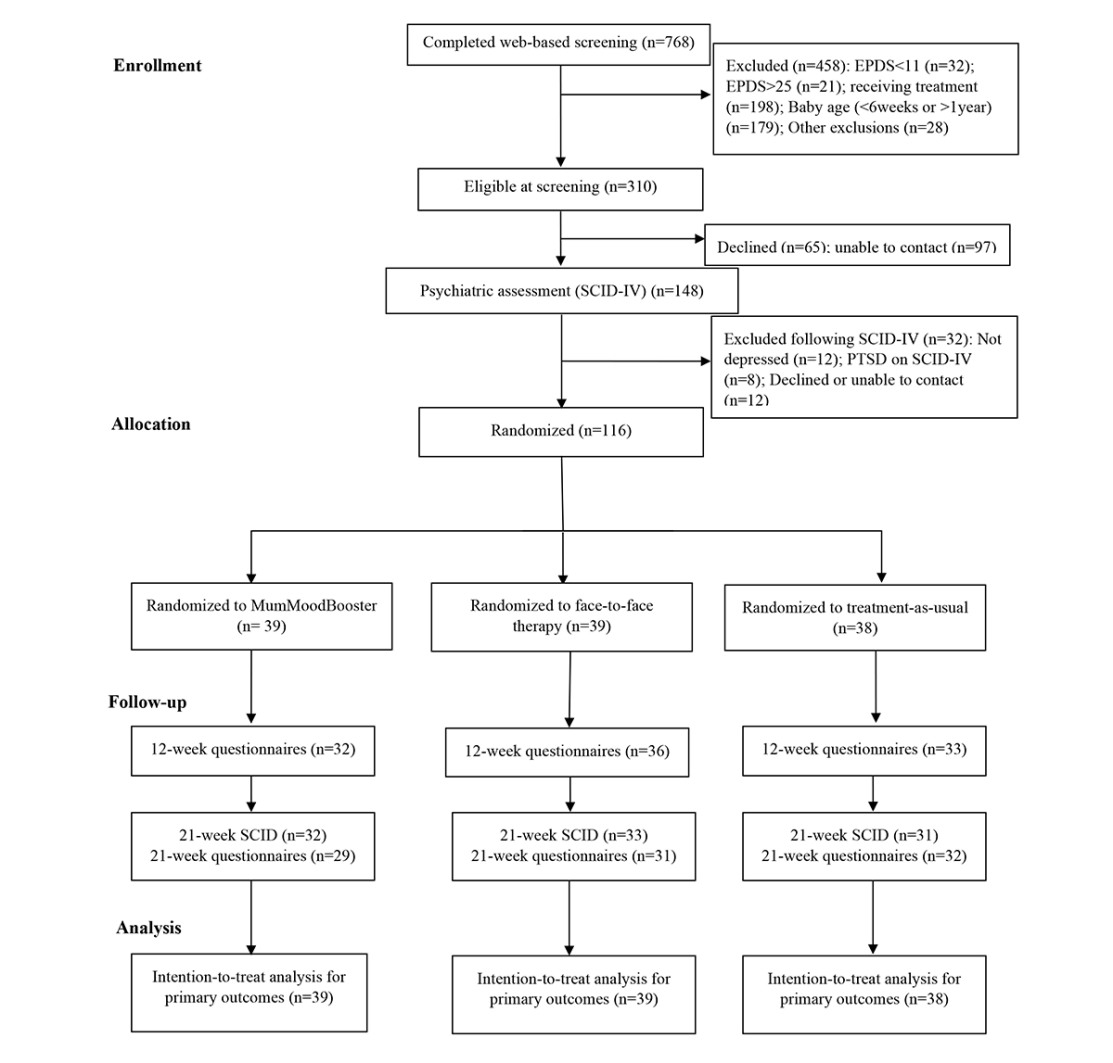
Participant flow through the study. EPDS: Edinburgh Postnatal Depression Scale; PTSD: posttraumatic stress disorder; SCID-IV: Structured Clinical Interview for the DSM-IV.

A total of 116 participants were randomized during the study period. Figure 1 presents a CONSORT chart of participant flow through the trial.

### Participant Characteristics

A range of primiparous and multiparous, high-income and low-income women were included (see participant baseline characteristics in Table 2). On average, participants were aged 32.1 years (SD 4.7); 66.4% (77/116) of participants were married, and regarding the highest level of education, 12.2% (14/115) completed high school, 26.1% (30/115) had completed a bachelor’s degree, and 34.8% (40/115) had completed graduate or postgraduate programs. The average infant age was 26.1 (SD 14.5) weeks. Chi-square tests and one-way analysis of variance were used to examine baseline demographic characteristics by condition. No group differences were found, confirming that randomization produced initially equivalent groups.

**Table 2:** Participant and baby characteristics by study condition (N=116)^a^.

Participant characteristics				MMB^b^ (n=39)		FTF-CBT^c^ (n=39)		TAU^d^ (n=38)
Age (years), mean (SD)				30.8 (4.3)		32.2 (5.3)		31.9 (4.2)
**Number of children,** **n** **(%)**								
	1			20 (51)		19 (49)		16 (42)
	2			17 (44)		15 (38)		16 (42)
	3			2 (5)		3 (8)		5 (13)
	≥4			0 (0)		2 (5)		1 (3)
**Marital status,** **n** **(%)**								
	Married			27 (69)		26 (67)		24 (63)
	De facto			12 (31)		10 (26)		11 (29)
	Separated			0 (0)		1 (3)		2 (5)
	Single			0 (0)		2 (5)		1 (3)
**Highest education,** **n** **(%)**								
	**Completed high school**							
		No	1 (3)		1 (3)		1 (3)	
		Yes	4 (10)		6 (16)		4 (11)	
	Certificate or apprenticeship			7 (18)		7 (18)		8 (21)
	Advanced diploma			4 (10)		1 (3)		1 (3)
	Bachelors			7 (18)		12 (32)		11 (29)
	Graduate			7 (18)		5 (13)		4 (11)
	Postgraduate			9 (23)		6 (16)		9 (24)
**Annual household income Aus $ (US $), n (%)**								
	>20,000 (14,800)			0 (0)		1 (3)		0 (0)
	20,001-40,000 (14,801-29,600)			0 (0)		6 (15)		3 (8)
	40,001-60,000 (29,601-44,400)			4 (10)		3 (8)		5 (13)
	60,001-80,000 (44,401-59,200)			8 (21)		5 (13)		7 (18)
	≥80,001 (59,201)			24 (62)		21 (54)		22 (58)
	Prefer not to disclose			3 (8)		3 (8)		1 (3)
Prior counseling, n (%)				26 (67)		26 (67)		20 (53)
Taken depression medication, n (%)				9 (23)		15 (38)		7 (18)
**Baby characteristics**								
	Age (weeks), mean (SD)			28.5 (14.1)		31.3 (16.1)		28.4 (13.7)
	Female, n (%)			25 (64)		24 (62)		18 (47)

^a^Column totals in percentages may not sum to 100; values in percentages are rounded upwards to the nearest full integer.

^b^MMB: MumMoodBooster.

^c^FTF-CBT: face-to-face cognitive behavioral therapy.

^d^TAU: treatment as usual.

### Missing Data and Attrition

Failure to complete scheduled assessments (attrition from the study) was 12.9% (15/116) at the 12-week assessment and 20.7% (24/116) at the 21-week assessment (Figure 1). Analysis indicated that attrition at these times was not related to study condition (*P*=.57), any of the demographic characteristics in Table 1 (all *P*>.22), or baseline measures of the primary or secondary outcomes (all *P*>.07).

### Engagement in MMB Program

Of the women allocated to the MMB intervention, 85% (33/39) completed ≥3 MMB sessions, and 72% (28/39) viewed all 6 MMB sessions. The MMB program was not visited by 3% (1/39) of participants. Of the women who visited MMB at least once, the mean number of sessions viewed was 5.6 (SD 1.7), and the mean number of visits was 15.6 (SD 8.7). The mean total time spent using MMB averaged 230 (range 2-440) minutes. The mean number of library articles accessed was 3.5 out of a possible 8, and the mean number of activities accessed was 15 out of 63. Approximately 39% (15/39) of participant partners accessed the partner support website.

### Engagement in FTF-CBT

Of the women offered FTF-CBT, 62% (24/39) completed ≥3 sessions, 51% (20/39) finished ≥6 sessions, and 46% (18/39) completed all 10 sessions. Approximately 31% (12/39) of women did not attend any FTF sessions. The mean number of FTF-CBT sessions attended for all 39 women was 5.5 (SD 4.5).

### Primary Outcomes

At baseline, all women in all 3 groups had a *DSM-IV* diagnosis of either major or minor depression; 92.2% (107/116) of the sample had a major depression diagnosis. Table 3 shows the change in the profile of depressive diagnostic status from baseline to the 21-week follow-up. On the basis of the observed data available at the 21-week follow-up assessment, the FTF-CBT group was 56.6% less likely to have a diagnosis of major or minor depression relative to the TAU group. The MMB group was 47.7% less likely to have a diagnosis of major or minor depression relative to the TAU group. Remission rates in the FTF-CBT and MMB conditions were not significantly different from each other (*χ*^2^_1_=0.1, *P*=.81). Table 4 provides descriptive statistics for the primary continuous outcome measures for each of the 3 conditions at baseline, 12 weeks, and 21 weeks, and Table 5 provides descriptive statistics for the secondary outcome measures.

**Table 3:** Diagnostic status of participants from baseline to the 21-week follow-up (N=116).

Conditions	Baseline, n (%)			21-week follow-up, n (%)			
	Major	Minor	Major		Minor	Lost to follow-up	Remission^a^
MMB^b^ (n=39)	36 (92)	3 (8)	4 (10)		3 (8)	7 (18)	25 (78)
FTF-CBT^c^ (n=39)	35 (90)	4 (10)	5 (13)		1 (3)	6 (15)	27 (82)
TAU^d^ (n=38)	36 (95)	2 (5)	12 (32)		1 (3)	7 (18)	18 (58)

^a^Remission was defined as the absence of a Structured Clinical Interview for the DSM-IV diagnosis for major or minor depression at the follow-up interview and excludes cases lost to follow-up.

^b^MMB: MumMoodBooster.

^c^FTF-CBT: face-to-face cognitive behavioral therapy.

^d^TAU: treatment as usual.

**Table 4:** Descriptive statistics for continuous primary outcomes.

Primary outcome			Baseline, mean (SD)		12-week, mean (SD)		21-week, mean (SD)
**Depressive symptoms (BDI-II)^a,b^**							
	MMB^c^	28.10 (7.91)		11.63 (8.96)		8.70 (6.92)	
	FTF-CBT^d^	27.18 (9.95)		21.36 (12.15)		15.00 (10.71)	
	TAU^e^	29.97 (8.76)		18.85 (10.16)		17.41 ()11.51	
**Anxiety symptoms (DASS-21)^f,g^**							
	MMB	8.87 (7.02)		1.81 (2.61)		2.69 (4.29)	
	FTF-CBT	9.23 (7.31)		8.11 (8.01)		4.45 (5.45)	
	TAU	8.58 (7.85)		4.73 (5.22)		5.19 (5.52)	

^a^BDI-II: Beck Depression Inventory, revised.

^b^BDI-II categories: minimal depression=0-13, mild depression=14-19, moderate depression=20-28, and severe depression=29-63.

^c^MMB: MumMoodBooster.

^d^FTF-CBT: face-to-face cognitive behavioral therapy.

^e^TAU: treatment as usual.

^f^DASS-21: Depression Anxiety Stress Scale, 21-item.

^g^DASS-21 categories: normal=0-7, mild=8-9, moderate=10-14, severe=15-19, and extremely severe=≥20.

**Table 5:** Secondary outcomes.

Secondary outcome		Baseline, mean (SD)	12-week, mean (SD)	21-week, mean (SD)
**Perceived stress (DASS-21)^a,b^**				
	MMB^c^	20.67 (7.99)	10.88 (7.22)	8.55 (6.88)
	FTF-CBT^d^	18.72 (8.10)	16.94 (9.15)	12.39 (7.97)
	TAU^e^	20.16 (7.52)	14.67 (6.28)	13.25 (8.11)
**Marital functioning (DAS-7^f^)**				
	MMB	21.23 (5.22)	21.25 (4.56)	23.21 (6.56)
	FTF-CBT	19.90 (7.66)	21.39 (6.18)	21.07 (7.87)
	TAU	21.79 (6.66)	22.49 (6.13)	22.22 (6.41)
**CBT^g^ skills-behavioral activation (BADS^h^)**				
	MMB	55.69 (18.12)	45.06 (17.52)	42.93 (14.13)
	FTF-CBT	57.03 (20.14)	50.78 (15.19)	40.84 (11.13)
	TAU	58.44 (15.92)	52.36 (16.15)	48.53 (16.53)
**CBT skills-negative thinking (ATQ^i^)**				
	MMB	47.72 (25.69)	21.44 (21.40)	16.52 (18.02)
	FTF-CBT	45.77 (28.65)	36.25 (27.74)	24.55 (23.60)
	TAU	52.84 (25.70)	34.15 (24.62)	32.66 (27.11)
**Maternal Self-efficacy subscale (PSOC^j^)**				
	MMB	24.03 (6.58)	29.66 (7.39)	30.90 (7.87)
	FTF-CBT	25.61 (7.96)	26.44 (7.12)	29.71 (7.26)
	TAU	22.45 (7.21)	28.03 (7.20)	28.09 (7.83)

^a^DASS-21: Depression Anxiety Stress Scale, 21-item.

^b^DASS-21 categories: normal=0-14, mild=15-18, moderate=19-25, severe=26-33, and extremely severe=≥34.

^c^MMB: MumMoodBooster.

^d^FTF-CBT: face-to-face cognitive behavioral therapy.

^e^TAU: treatment as usual.

^f^DAS-7: Dyadic Adjustment Scale.

^g^CBT: cognitive behavioral therapy.

^h^BADS: Behavioral Activation for Depression Scale.

^i^ATQ: Automatic Thoughts Questionnaire.

^j^PSOC: Parenting Sense of Competence Scale.

### Growth Models

Parameter estimates from the growth models for each a priori comparison are provided in Tables 6-8. These include parameter estimates for intercept, condition, time, and condition×time interaction.

**Figure 2:**
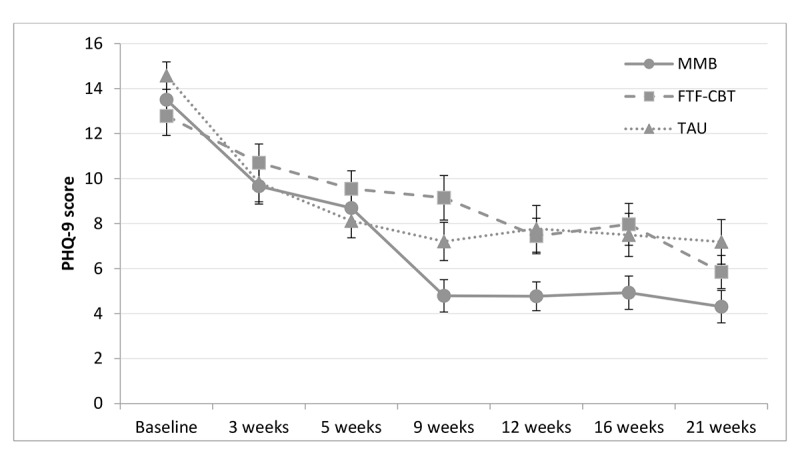
Change from baseline to 21-week follow-up for the MMB, FTF-CBT and TAU groups on the PHQ-9. Mean values are observed values plotted ±1 SE. PHQ-9 score ranges: minimal or none=0-4, mild=5-9, moderate=10-14, moderately severe=15-19, and severe ≥20. FTF-CBT: face-to-face cognitive behavioral therapy; MMB: MumMoodBooster; PHQ-9: Patient Health Questionnaire–9; TAU: treatment as usual.

In Table 6, the intercept reflects the model-implied estimated baseline depressive score (PHQ-9) for the TAU group (29.02). The *t* value and *P* value indicate that the intercept is significantly different from 0 (*P*<.001). The condition parameter (−2.154) indicates that the MMB condition had an estimated baseline score that was 2.15 points lower than the TAU group. The *t* value and *P* value indicate that the lower baseline depressive score for the MMB condition was not statistically significant (*P*=.24). The time parameter (−0.614) indicates that the TAU group had an estimated linear decrease in depressive symptom score of 0.61 points for each week through the 21-week follow-up assessment. The *t* value and *P* value indicate that the weekly linear decrease for the TAU group is significantly different from 0 (*P*<.001). The condition×time interaction term (−0.3029) indicates that the MMB group decreased an additional 0.30 points each week through the 21-week follow-up. The *t* value and *P* value indicate that the greater decrease over time for the MMB group relative to the TAU group was statistically significant (*P*=.01). Figure 2 shows the trajectory of the PHQ-9 scores for each condition over the course of the study. Significant condition×time effects were also found for anxiety and perceived stress (Table 6), indicating that MMB was statistically superior to TAU in reducing symptoms of depression, anxiety, and stress from baseline to the 21-week follow-up.

**Table 6:** Parameter estimates from growth models for MumMoodBooster (MMB) versus treatment as usual (TAU).

Outcomes and growth model parameters			Parameter (SE)		*t* test^a^ (*df*)		*P* value
**Depressive symptoms**							
	Intercept	29.024 (1.301)		22.31 (75)		<.001	
	Condition	−2.154 (1.831)		−1.18 (75)		.24	
	Time	−0.614 (0.088)		−7.01 (124)		<.001	
	Condition×time	−0.329 (0.126)		−2.61 (124)		.01	
**Anxiety symptoms**							
	Intercept	8.144 (1.129)		7.21 (75)		<.001	
	Condition	0.012 (1.588)		0.01 (75)		.99	
	Time	−0.163 (0.052)		−3.15 (124)		.002	
	Condition×time	−0.148 (0.074)		−2.01 (124)		.05	
**Perceived stress**							
	Intercept	19.799 (1.159)		17.08 (75)		<.001	
	Condition	0.186 (1.631)		0.11 (75)		.91	
	Time	−0.323 (0.068)		−4.78 (124)		<.001	
	Condition×time	−0.270 (0.097)		−2.78 (124)		.006	
**Marital functioning**							
	Intercept	21.906 (0.930)		23.55 (75)		<.001	
	Condition	−0.898 (1.308)		−0.69 (75)		.50	
	Time	0.030 (0.042)		0.72 (124)		.47	
	Condition×time	0.068 (0.060)		1.13 (124)		.26	
**CBT^b^ skills: behavioral activation**							
	Intercept	58.158 (2.651)		21.94 (75)		<.001	
	Condition	−3.258 (3.728)		−0.87 (75)		.39	
	Time	−0.466 (0.137)		−3.40 (124)		.001	
	Condition×time	−0.134 (0.197)		−0.68 (124)		.50	
**CBT skills: negative thinking**							
	Intercept	51.106 (3.992)		12.80 (75)		<.001	
	Condition	−5.410 (5.614)		−0.96 (75)		.34	
	Time	−0.996 (0.185)		−5.40 (124)		<.001	
	Condition×time	−0.408 (0.265)		−1.54 (124)		.13	
**Maternal Self-efficacy subscale (PSOC^c^)**							
	Intercept	23.021 (1.092)		21.08 (75)		<.001	
	Condition	1.364 (1.535)		0.89 (75)		.38	
	Time	0.274 (0.059)		4.65 (124)		<.001	
	Condition×time	0.077 (0.085)		0.92 (124)		.36	

^a^Tests were two-tailed.

^b^CBT: cognitive behavioral therapy.

^c^PSOC: Parenting Sense of Competence Scale.

Tables 7 and 8 show the results for the other a priori comparisons: FTF-CBT versus TAU and MMB versus FTF-CBT. Although none of the condition×time effects were significant for the FTF-CBT versus TAU comparison (Table 7), the MMB versus FTF-CBT comparison revealed that MMB performed significantly better than FTF-CBT in reducing depressive symptoms and perceived stress (Table 8).

The effect size (*d*) for all the condition×time interaction terms is presented in Table 9. Compared with TAU, the MMB group reported a large reduction in depressive symptoms (*d*=−0.83) and medium-sized reductions in anxiety (*d*=−0.42) and perceived stress (*d*=−0.73). Compared with the FTF-CBT condition, MMB participants reported large reductions in both depressive symptoms (*d*=−0.98) and perceived stress (*d*=−0.89). The TAU and FTF-CBT conditions did not differ significantly in any of the outcome measures.

**Table 7:** Parameter estimates from growth models of face-to-face cognitive behavioral therapy (CBT) versus treatment as usual.

Outcomes and growth model parameters			Parameter (SE)		*t* test^a^ (*df*)		*P* value
**Depressive symptoms**							
	Intercept	29.047 (1.533)		18.94 (75)		<.001	
	Condition	−1.733 (2.155)		−0.80 (75)		.42	
	Time	−0.611 (0.094)		−6.52 (124)		<.001	
	Condition×time	0.110 (0.132)		0.83 (124)		.41	
**Anxiety symptoms**							
	Intercept	8.140 (1.215)		6.70 (75)		<.001	
	Condition	1.389 (1.707)		0.81 (75)		.42	
	Time	−0.155 (0.054)		−2.90 (124)		.004	
	Condition×time	−0.012 (0.076)		−0.16 (124)		.88	
**Perceived stress**							
	Intercept	19.799 (1.218)		16.26 (75)		<.001	
	Condition	−0.705 (1.711)		−0.41 (75)		.68	
	Time	−0.314 (0.060)		−5.23 (124)		<.001	
	Condition×time	0.068 (0.085)		0.80 (124)		.42	
**Marital functioning**							
	Intercept	21.911 (1.138)		19.26 (75)		<.001	
	Condition	−1.874 (1.599)		−1.17 (75)		.25	
	Time	0.029 (0.050)		0.57 (124)		.57	
	Condition×time	−0.012 (0.070)		−0.17 (124)		.87	
**CBT skills: behavioral activation**							
	Intercept	−0.420 (4.015)		−0.10 (75)		.92	
	Condition	−0.420 (4.015)		−0.10 (75)		.92	
	Time	−0.459 (0.142)		−3.24 (124)		.002	
	Condition×time	−0.217 (0.200)		−1.09 (124)		.28	
**CBT** **skills: negative thinking**							
	Intercept	51.091 (4.272)		11.96 (75)		<.001	
	Condition	−4.741 (6.002)		−0.79 (75)		.43	
	Time	−0.989 (0.204)		−4.84 (124)		<.001	
	Condition×time	0.075 (0.288)		0.26 (124)		.80	
**Maternal Self-efficacy subscale (PSOC^c^)**							
	Intercept	23.015 (1.204)		19.11 (75)		<.001	
	Condition	2.366 (1.692)		1.40 (75)		.17	
	Time	0.278 (0.066)		4.19 (124)		<.001	
	Condition×time	−0.096 (0.093)		−1.03 (124)		.31	

^a^Tests were two-tailed.

^b^PSOC: Parenting Sense of Competence Scale.

**Table 8:** Parameter estimates from growth models for MumMoodBooster versus face-to-face cognitive behavioral therapy (CBT).

Outcomes and growth model parameters			Parameter (SE)		*t* test^a^ (*df*)		*P* value
**Depressive symptoms**							
	Intercept	27.373 (1.446)		18.93 (76)		<.001	
	Condition	−0.491 (2.047)		−0.24 (76)		.81	
	Time	−0.525 (0.086)		−6.12 (126)		<.001	
	Condition×time	−0.418 (0.123)		−3.39 (126)		.001	
**Anxiety symptoms**							
	Intercept	9.567 (1.108)		8.64 (76)		<.001	
	Condition	−1.419 (1.568)		−0.90 (76)		.37	
	Time	−0.182 (0.057)		−3.17 (126)		.002	
	Condition×time	−0.129 (0.082)		−1.57 (126)		.12	
**Perceived stress**							
	Intercept	19.109 (1.236)		15.46 (76)		<.001	
	Condition	0.879 (1.750)		0.50 (76)		.62	
	Time	−0.252 (0.064)		−3.96 (126)		<.001	
	Condition×time	−0.341 (0.092)		−3.73 (126)		<.001	
**Marital functioning**							
	Intercept	20.074 (0.977)		20.55 (76)		<.001	
	Condition	0.932 (1.383)		0.67 (76)		.50	
	Time	0.004 (0.044)		0.08 (126)		.94	
	Condition×time	0.097 (0.064)		1.52 (126)		.13	
**CBT skills: behavioral activation**							
	Intercept	57.725 (2.917)		19.79 (76)		<.001	
	Condition	−2.800 (4.130)		−0.68 (76)		.50	
	Time	−0.695 (0.145)		−4.78 (126)		<.001	
	Condition×time	0.082 (0.207)		0.40 (126)		.69	
**CBT skills: negative thinking**							
	Intercept	46.395 (4.291)		10.81 (76)		<.001	
	Condition	−0.640 (6.073)		−0.11 (76)		.92	
	Time	−0.935 (0.204)		−4.58 (126)		<.001	
	Condition×time	−0.499 (0.293)		−1.71 (126)		.09	
**Maternal self-efficacy subscale (PSOC^b^)**							
	Intercept	25.375 (1.152)		22.03 (76)		<.001	
	Condition	−0.987 (1.630)		−0.61 (76)		.55	
	Time	0.188 (0.058)		3.24 (126)		.002	
	Condition×time	0.162 (0.084)		1.94 (126)		.06	

^a^Tests were two-tailed.

^b^PSOC: Parenting Sense of Competence Scale.

**Table 9:** Effect sizes (d) and significance levels for condition×time differences from mixed-effects growth models^a^.

Outcome measures		MMB^b^ vs TAU^c^		FTF-CBT^d^ vs TAU		MMB vs FTF-CBT	
		*d*	*P* value	*d*	*P* value	*d*	*P* value
Depressive symptoms		–0.83	.01^e^	0.25	.41	–0.98	.001^e^
Anxiety symptoms		–0.42	.05^e^	–0.03	.88	–0.38	.12
Perceived stress		–0.73	.006^e^	0.18	.42	–0.89	<.001^e^
Marital functioning		0.2	.26	–0.03	.87	0.29	.13
**CBT^f^ skills**							
	Behavioral activation	–0.16	.50	–0.25	.28	0.09	.69
	Negative thinking	–0.33	.13	0.06	.80	–0.40	.09
Maternal self-efficacy		0.24	.36	–0.27	.31	0.47	.06

^a^For each comparison, the first group is the reference group.

^b^MMB: MumMoodBooster.

^c^TAU: treatment as usual.

^d^FTF-CBT: face-to-face cognitive behavioral therapy.

^e^Significant effects at *P*<.05.

^f^CBT: cognitive behavioral therapy.

### Use of Other Treatments

Of the 79.3% (92/116) of participants who responded to this questionnaire, approximately 19% (6/32) of participants in the TAU group and 6% (2/31) of participants in the FTF-CBT group reported using depression medication. None of the MMB participants reported using medication. Approximately 3% (1/31) of women in the FTF-CBT group, 7% (2/29) of women in the MMB group, and 6% (2/32) of women in the TAU group reported having taken part in group therapy. Approximately 23% (7/31), 21% (6/29), and 13% (4/32) of women in the FTF-CBT, MMB, and TAU conditions, respectively, reported using self-help books, and 2 women each in the MMB and TAU groups reported having used acupuncture or hypnotherapy.

### Helpfulness and Satisfaction

Participants in the 2 active conditions (FTF-CBT and MMB) rated the helpfulness of their treatment on a rating scale of 0 to 3. Compared with those in the MMB group (mean 2.31, SD 0.89), participant ratings in the FTF-CBT group (mean 2.84, SD 0.38) were significantly higher (*P*=.02).

MMB participants rated the helpfulness of the coach calls an average of 2.56 (SD 0.67) out of a possible 3 points. MMB participants further rated their satisfaction (from 0 to 3) with the following features of the internet program: mood tracking (mean 2.06, SD 0.91), pleasant activities (mean 2.28, SD 0.85), strategies to reduce negative thinking (mean 2.26, SD 1.03), strategies to increase positive thinking (mean 2.19, SD 0.91), partner support program (mean 1.19, SD 1.02), library articles (mean 1.96, SD 1.04), and videos (mean 1.87, SD 1.07).

## Discussion

Existing research confirms that PND can be successfully treated by internet-delivered psychological interventions [51]. However, no previously published study has made a direct comparison of the efficacy of such interventions with traditional FTF treatment in a clinically diagnosed population.

### Principal Findings

In a sample of postpartum women, 92.2% (107/116) of whom had a major depression diagnosis and were in the moderate-to-severe range for depression symptoms on the BDI-II, we found that the MMB internet-delivered treatment program performed at least as well as FTF-CBT on remission from a diagnosed depressive episode at 21 weeks. Moreover, MMB was also significantly more effective than FTF-CBT in reducing depression symptom severity and perceived stress over time. Compared with TAU, MMB was significantly more effective in reducing depression, anxiety, and perceived stress. At follow-up, depression and anxiety symptom scores for MMB participants were approximately 50% lower than those observed in the FTF-CBT and TAU conditions.

These results are broadly consistent with the literature that delivery of psychological treatments via the internet can be as effective as traditional FTF approaches for treating depression and anxiety [26-28,88,89]. Moderate-to-large effects are generally reported [90], although it should be noted that in many studies, waitlist controls and TAU are the typical comparators. The findings also parallel the results from our previous RCT [43], which showed a significantly larger reduction in depression symptoms in the MMB condition compared with TAU.

The use of coaches as an adjunct to the MMB program may have contributed to the relatively low attrition in this condition and enhanced the effectiveness of the MMB program. This is consistent with our previous trials of MMB [43,91] and with current evidence suggesting that fully automated treatments can be enhanced through support, whether guided by a technician or therapist via phone, email, or SMS text messages [19,29,31]. For example, Karyotaki et al [92] conducted a meta-analysis of a range of guided internet interventions for depression and found significantly better efficacy for guided interventions compared with fully automated interventions. The current evidence base confirms that self-guided interventions do produce benefits; however, guided support seems to enhance the efficacy of many internet interventions [25,27,93].

### Strengths

This study has a number of strengths. First, this RCT produced very similar effect sizes for reductions in depression and anxiety symptoms and the rate of session attendance and program adherence compared with those we observed in our 2 previous trials of MMB [42,43]. In our earlier RCT [43], MMB produced a 4-fold difference in remission compared with TAU as measured at the 12-week follow-up. In this study, follow-up occurred considerably later (at 21 weeks). Remission rates in the MMB condition were still found to be similar to those in our previous RCT [43] and superior to TAU, albeit with a smaller between-group difference in depression remission rate than at 12 weeks in the previous trial. This may indicate that the gains made during the program were maintained over this longer follow-up period. This difference between our previous RCT (measured at 12 weeks) and in our current RCT (measured at 21 weeks) is perhaps not unexpected, given that meta-analyses suggest considerable spontaneous remission rates even in untreated individuals over longer time frames [7].

Importantly, this research provides the first direct comparison of internet-delivered psychological treatment for perinatal depression with evidence-based FTF treatment. This was achieved in a sample of women clinically diagnosed with depressive disorders rather than in a sample of women with a mix of subclinical symptoms assessed using screening tools as in many existing studies.

Furthermore, the rate at which participants accessed other treatments outside of those provided in the trial was low and appeared similar in the 2 active treatment groups. However, despite the higher use of *other supports* found in the TAU group, the participants assigned to the MMB condition reported greater reductions in depression and anxiety symptom scores.

Our use of a 3-arm RCT that included TAU and intention-to-treat analyses gives us further confidence in interpreting these results. As such, this study extends the literature showing promising results from trials of internet-based treatments for PND [50,51].

The FTF-CBT intervention was primarily provided by practicing psychologists in the community, making the comparison as close an approximation as possible to a real-world treatment option. In Australia, FTF-CBT is 1 of the 2 main treatments that postnatal women with depression are likely to be offered (the other being antidepressant medication).

As our results showed that MMB was at least comparable with traditional FTF-CBT in efficacy, its more widespread implementation could potentially increase the reach of treatment to benefit women with PND not currently accessing traditional services because of geographic or social isolation or through a preference for anonymous, nonclinical settings.

### Limitations

The study has a number of limitations that should be noted, some of which are common to most RCTs. Our observed 21-week outcomes may have reflected some cases in which relapse occurred after treatment completion, perhaps in both of the active treatments as well as some spontaneous recovery over time in the TAU group. Participants in this study agreed to be assigned to any of the 3 possible experimental conditions; therefore, they may be a particular subset of the population and not representative of postnatal women with depression as a whole. It is possible that our study results may not generalize to women who would have preferred to use only the internet intervention (MMB) or alternatively only attend the clinic-based FTF-CBT treatment. Furthermore, given the nature of the intervention, the participants could not be blinded beyond the point of treatment allocation. Finally, the limited sample size was not sufficiently powered to examine subgroup differences or the moderating effects of baseline participant characteristics.

### Future Directions

Emerging evidence suggests that the COVID-19 pandemic is seeing substantial increases in perinatal mental health difficulties [94,95] concurrent with mandatory restrictions on population movement. Therefore, access to FTF psychological services may be problematic in many locations, and effective, accessible internet interventions for perinatal depression are likely to be highly relevant. There is increasing recognition that the impact of real-world implementation of internet mental health interventions and their cost-effectiveness [96] depends upon how treatments are configured and integrated into the mental health treatment landscape as a whole [97,98].

Various options for deploying digital delivery as part of a *blended* or guided approach that combines clinical therapy or counseling with internet program content show promise [99-101], with relevance across a range of diagnoses [102]. Systematic reviews suggest that programs that combine traditional and digital approaches can achieve clinically meaningful effects on depression and anxiety outcomes [103], and based on current evidence [96,104,105], it seems reasonable to infer that internet CBT could also be a cost-effective complement to the support provided to women with PND in primary care. An important potential advantage is that internet CBT can be commenced more or less immediately, with reduced or no waiting time. This could facilitate very early intervention if, for example, the intervention program were linked to routine depression screening in the very early postpartum period.

Evaluation is also warranted for the stepped-care model or adaptive treatment strategies for moderate depression and anxiety symptoms that offer a standalone internet intervention as an initial treatment option [106,107]. Future research is needed to examine program effectiveness and implementation scenarios associated with offering standalone internet PND interventions that do not include human guidance. For example, the scalability and sustainability of supported internet CBT depend at least in part on the resources available to such programs, so that unguided programs of comparable efficacy may be a useful alternative in many service delivery contexts [34]. Important questions also remain around issues such as patient adherence and the specific health care population from which users of internet psychological interventions are drawn, both in the real-world setting [108,109] and in efficacy RCTs [110]. We found some evidence that session attendance in the MMB condition may have been somewhat higher than in FTF-CBT. Finally, variations of (or complements to) MMB deserve additional research attention, including internet depression treatment programs for pregnant women as well as for new fathers who have so far been largely neglected.

### Conclusions

The current trial replicated key findings of prior MMB studies, providing further evidence that internet-delivered treatment for postnatal women with depression is a viable treatment option. These findings are broadly consistent with the notion that speed of access, anonymity, and convenience may be specific advantages of internet-delivered treatment for women who prefer not to engage in traditional treatment modalities. Notably, this study also provides empirical evidence that participants assigned to the MMB condition report greater treatment gains compared with those assigned to the TAU and FTF-CBT conditions.

## Appendix

Multimedia Appendix 1 CONSORT e-HEALTH checklist (V 1.6.1).

## Abbreviations

ATQ: Automatic Thoughts Questionnaire
BDI-II: Beck Depression Inventory, revised
CBT: cognitive behavioral therapy
DASS-21: Depression Anxiety Stress Scale, 21-item
*DSM-IV*: *Diagnostic and Statistical Manual for Mental Disorders, Fourth Edition*
FTF: face-to-face
GP: general practitioner
MMB: MumMoodBooster
PHQ-9: Patient Health Questionnaire, 9-item
PND: postnatal depression
RCT: randomized controlled trial
SCID-IV: Structured Clinical Interview for the DSM-IV
TAU: treatment as usual
